# QEEG in affective disorder: about to be a biomarker, endophenotype and predictor of treatment response

**DOI:** 10.1016/j.heliyon.2018.e00741

**Published:** 2018-08-22

**Authors:** Sermin Kesebir, Ahmet Yosmaoğlu

**Affiliations:** Üsküdar University, NPİstanbul Brain Hospital, İstanbul, Turkey

**Keywords:** Clinical psychology, Psychiatry, Medical imaging, Neurology, Neuroscience

## Abstract

QEEG is a relatively easy to apply, cost effective method among many electrophysiologic and functional brain imaging techniques used to assess individuals for diagnosis and determination of the most suitable treatment. Its temporal resolution provides an important advantage. Many specific EEG indicators play a role in the differential diagnosis of neuropsychiatric disorders. QEEG has advantages over EEG in the dimensional approach to symptomatology of psychiatric disorders. The prognostic value of EEG has a long history. Slow wave EEG rhythm has been reported as a predictor and measure of clinical improvement under ECT. The induction level in delta band activity predicts the long term effect of ECT. Current studies focus on the predictive power of EEG on response to pharmacotherapy and somatic treatments other than ECT. This paper discusses either QEEG can be a biomarker and/or an endophenotype in affective disorders, if it has diagnostic and prognostic value and if it can contribute to personalized treatment design, through a review of relevant literature.

## 1 Introduction

EEG reflects the electrical activity of the brain by recording through the scalp presynaptic and postsynaptic potentials arising from simultaneous firing of a group of neurons (Leuchter et al., 1999). Cortical discharges of 1.5 mV amplitude are amplified and decomposed with Fourier transformation. EEG, as opposed to fMRI and PET, reflects neuronal activity directly rather than using indirect measures such as blood deoxygenation and glucose utilization. Its temporal resolution is greater than both.

QEEG can be conceptualized as the numeric interpretation of EEG waves and brain mapping as the two dimensional visualization of this interpretation. LORETA (low resolution brain electromagnetic tomography) provides a three dimensional evaluation. It is generated by an algorithm for intracortical EEG computations. Spectral power density provides an estimate of radial current flow in localized brain regions. It also eliminates the confusion resulting from differences in reference electrodes. Therefore, this new approach is reported to yield more direct results (Tenke and Kayser, 2005).

### 1.1 Personalised medicine

Descriptive diagnostic systems ignore neurobiological heterogeneity. The most crucial result of the STAR-D trial is the demonstration of the limited efficacy of pharmacotherapies and psychotherapies, especially in the long term (Rush et al., 2006). Thus situated, lack of effective treatment options makes it a requirement to try personalizing the existing treatment options so as to choose the best option for a specific individual. As a matter of fact, a single DSM diagnosis matches more than one treatment option.

### 1.2 Candidacy for a biomarker and endophenotype

Personalized medicine (NIMH Strategic Plan on Research Domain Criteria and termed Precision Medicine) is focused on biomarkers and endophenotypes. Biomarkers change among disease subtypes. Endophenotype is the heritable form of biomarker, which possesses genotypic and phenotypic information. Hitherto, personality and temperament questionnaires, cognitive function tests, neurophysiological tests, neurotransmitter metabolites, pharmacogenomics and pharmacometabolomics were suggested and studied as candidates. Though all proved distinctive, reliable and applicable to some extent, none gained firm ground in clinical practice.

Heritability of some QEEG parameters such as alpha peak frequency and alpha spectral power density was shown in twin and family studies. The heritability quotients of these two variables were determined as 81 and 79% respectively. There rates are superior to p300 amplitude and latency values previously determined as 60 and 51% respectively (Vogel, 1970). Alpha peak frequency was found to be 1.4 Hz slower in relation to COMT gene Val/Val genotype and was accepted as an endophenotype for treatment resistant depression. Also low-voltage alpha is associated with HTR3B, CRH-BP, GABA-B and BDNF Val66Met polymorphisms and heritability is reported to be 79–93% for these variables. Decrease in frontal alpha and theta activity, which has been conceptualized as impaired vigilance, is reported to predict response to antidepressants in depression. According to these studies nature and nurture act in tandem.

### 1.3 Diagnostic and prognostic value

Many EEG indicators have the capability to distinguish between neuropsychiatric conditions. This was described first for depression and ADHD, followed by bipolar affective disorder and dementia (Olbrich and Arns, 2013; Savitz et al., 2013). A cautionary point is that DSM's ‘impaired daily function’ criterion is crucial in order to decide whom to treat; like the distinction between an artist and a CEO with a similar degree of impulsivity or between a street dweller and a petty criminal.

### 1.4 Depressive disorder

Lemere remarked ‘the ability to produce a quality alpha wave is associated with the affective repertoire of the brain’ (1936). Increased or decreased alpha power density is a criterion for depression (Itil, 1983; Ulrich et al., 1984). Lieber and Newbury described two groups of depressive patients according to QEEG data in a 1988 study on 216 inpatients. The first group showed an increase in activity along with beta and/or slow wave whereas the second group showed an increase in slow wave activity. This data, in conjunction with the results of later research, can currently be interpreted as the first group having bipolar depression. QEEG's feasibility as a biomarker that can distinguish between unipolar and bipolar depression makes this interpretation noteworthy.

The prognostic value of EEG has a long history. Slow wave EEG rhythm has been reported as a predictor and measure of clinical improvement under ECT (Fink, 2010). The induction level in delta band activity predicts the long term effect of ECT (Volavka et al., 1972; Nobler et al., 2002).

Widespread slow wave activity is an index of antidepressant unresponsiveness. In a multicentered, large sample study that assesses treatment response for 8 weeks to escitalopram and venlafaxine with HAM-D, comparing cases with or without abnormal slowing of QEEG, treatment response rate to escitalopram was found to be 33% in cases with and 64% in cases without abnormal QEEG slowing; treatment response rate to venlafaxine was found to be 41% in cases with and 66% in cases without abnormal QEEG slowing (Arns et al., 2017). P3 and N1 latencies and amplitudes were differentiated in nonresponders in this study (van Dinteren et al., 2015). Response solely to sertraline was observed when the decrease in alpha peak frequency was taken as a criterion.

Increased theta band activity in rostral ACC activity predicts antidepressant response and yielded positive results in 19 of 23 studies (ES = 0.918) (Pizzagalli, 2011). The treatments used in the studies mentioned in this metaanalysis are SSRI, TCA, TMS and sleep deprivation. Pretreatment rACC theta activity represents a nonspecific prognostic marker of treatment outcome in major depressive disorder. In their double-blind, placebo-controlled multicentered study, Pizzagalli et al. (2018) demonstrated that higher rostral anterior cingulate cortex theta activity at both baseline and week 1 predicted greater improvement in depressive symptoms in patients treated with either SSRIs or placebo, even when sociodemographic and clinical variables were controlled. According to their findings, of the 39.6% variance in symptom change, only 8.5% was uniquely attributable to the rACC theta marker.

Bruder et al. (2008) have found pretreatment differences between SSRI responders and nonresponders regarding EEG alpha power or asymmetry. Treatment responders had greater alpha power than nonresponders and healthy subjects at occipital sites where alpha was most prominent. Responders showed less activity over right than left hemisphere, whereas nonresponders tended to show the opposite asymmetry. Neither alpha power nor asymmetry changed after treatment. According to their findings alpha power and asymmetry possessed reasonable positive predictive value but less negative predictive value.

Increased or decreased parietooccipital alpha, described as hyperstable vigilance, predicts the response only to dopaminergic antidepressants and stimulants (Pizzagalli et al., 2018). Hyperstable vigilance can be described as increased but undistractible and targeted vigilance. On the other hand serotonin and dopamine transporter availability during long-term antidepressant therapy does not differentiate responder and nonresponder unipolar patients (Cavanagh et al., 2006). There was no association between SERT and DAT availability. Alpha peak frequency is found to be slowed in depression unresponsive to TCA and also response rate to TMS is lower in these cases. In responsive cases alpha peak frequency is shown to be increased by 0.5 Hz at the end of the 4 th week (Arns et al., 2010). In a study using SPD mesures of median alpha value, positive predictive value was reported as 93.3% and specificity as 92.3% in 41 drug-free cases and no difference was found between SSRI ans SNRI use regarding prediction of treatment response (Tenke et al., 2011). Response to 6-week paroxetine treatment was predicted by gamma synchonization in QEEG correlated with HAM-D in 18 drug-free cases (Arıkan et al., 2018).

Discordance was defined as a severity variable in QEEG. It is the increase in relative activity while absolute activity is decreased. Cut off point is determined as 0.30. Although used frequently for theta and beta bands, it can be adapted to all other bands. Its validity is supported by PET studies and it is found to be correlated with low perfusion and metabolism. Discordance is an indicator of unresponsiveness to antidepressive treatment (Leuchter et al., 1999).

Prefrontal theta cordance was reported to predict response to venlafaxine treatment in the first week in treatment-resistant depression (Bares et al., 2008). The same researchers reported that prefrontal theta cordance can also predict response to bupropion augmentation treatment and TMS ( Bares et al., 2015). In the metaanalysis by Iosifescu et al. (2009) response prediction rate for SSRI, SNRI, TCA, TMU and ECT at the end of the first week was reported as 72–88%. In Spronk et al.'s (2011) regression model, better clinical outcome was characterized by a decrease in the amplitude of the Auditory Oddball N1 at baseline, impaired verbal memory performance was the best cognitive predictor, and raised frontal Theta power was the best EEG predictor of change in HAM-D scores. Arns et al. (2012) examined neurophysiological predictors of non-response to rTMS in depression. According to their results, non-responders were characterized by increased fronto-central theta EEG power, a slower anterior individual alpha peak frequency, a larger P300 amplitude, and decreased pre-frontal delta and beta cordance.

In a relatively old study visual average evoked responses to four intensities of light were studied in hospitalized depressed patients receiving placebo, d-amphetamine, l-amphetamine, lithium and d- and l-amphetamine combined with lithium (Buchsbaum et al., 1977). The amount of increase in evoked potential amplitude or amplitude/intensity slope seen with amphetamine was also significantly correlated with the amount of increase in activation or euphoria ratings with amphetamine administration. These effects were most prominent in the P100 component that we have previously found to differentiate bipolar and unipolar depressed patient groups (Koca and Kesebir, 2015). Several studies show that the response to selective serotonin reuptake inhibitors can be successfully predicted by using the loudness dependence of auditory evoked potentials (Juckel et al., 2007). Patients at the beginning of an antidepressant treatment who show an initially strong loudness dependence of auditory evoked potentials have a greater probability of responding to a serotonergic antidepressant, whereas patients with a weak loudness dependence will probably benefit more from a nonserotonergic agent.

Recent electrophysiological studies of emotional processing have provided new evidence of altered laterality in depressive disorders. EEG alpha asymmetry at rest and during cognitive or emotional tasks are consistent with reduced left prefrontal activity, which may impair downregulation of amygdala response to negative emotional information. Dichotic listening and visual hemifield findings for non-verbal or emotional processing have revealed reduced right-lateralized responsivity in depressed patients to emotional stimuli in occipitotemporal or parietotemporal cortex (Bruder et al., 2017a, b). Individual differences of right-left brain function are related to diagnostic subtype of depression, comorbidity with anxiety disorders, and clinical response to antidepressants or cognitive behavioral therapy. In another study, responders to CT showed twice the mean right ear advantage in dichotic fused words performance than non-responders. Patients with a right ear advantage greater than healthy controls had an 81% response rate to CT, whereas those with performance lower than controls had a 46% response rate (Bruder et al., 2017a, b). Individuals with a right ear advantage, indicative of strong left hemisphere language dominance, may be better at utilizing cognitive processes and left frontotemporal cortical regions critical for success of CT for depression.

The predictive power of QEEG on treatment response does not seem to be affected by gender (Arns et al., 2016). Were there findings on the contrary, they would be interpreted as originating from temperamental gender differences. In fact, among affective temperament types suggested as endophenotypes for affective disorders, depressive, cyclothymic and anxious temperaments are more common in females whereas hyperthymic and irritable temperaments are more commonly seen in males (Vahip et al., 2005).

While studies so far focused on the prediction of treatment response, the more important question is whether QEEG can predict recovery from depression. Tenke et al. (2015) asserted that the predictive capability of QEEG is lower for recovery and explained this with the failure of the previous treatment. There are case reports indicating that prefrontal theta cordance can rule out placebo response and dissimualtion and can predict manic shift (Bares et al., 2007; Leuchter et al., 2002; Kopecek et al., 2008). Our interpretation is that the condition called fade out of response to antidepressant is seen more frequently in bipolar depression and lower prediction rate may be due to these cases being in the bipolar spectrum.

### 1.5 Bipolar disorder

Should bipolar disporder be defined on the level of neuronal activity, incongruence between prefrontal and limbic activities must be mentioned. It is dysfunctional connectivity (Güven et al., 2015). It is the imparment in early P50 and N100 neural response. IFG activity decreases in mania whereas it returns to normal in depression and euthymia. Limbic activity is increased regardless of mood periods (Chen et al., 2011).

Overactivation in medial temporal lobe distinguishes bipolar cases from cases of schizophrenia in tasks stimulating emotion and memory (Whalley et al., 2012). It is shown to distinguish bipolar cases from unipolar cases and bipolar disorder type I cases from bipolar disorder type 2 cases in small-sample studies (Diler et al., 2013; Grotegard et al., 2013; Marchand et al., 2013; Gürdal and Kesebir, 2015).

Manic episode exhibits more frequency variation than depressive episode. It is frequently seen as increased beta activity and left dominant frontal alpha asymmetry. Similarly, frontal asymmetry is observed to persist in hipomania and, in a trait-based manner, in periods of remission (El-Badri et al., 2001). In a longitudinal follow up study on bipolar cases, severity of manic symptoms predicted worse insight without direct or moderating influences of global cognitive abilities (Depp et al., 2014). However, it must be stressed that the findings of this study are limited to six months of follow up.

Lithium is an agent which corrects the frontal function in the fourteenth day, which is minimally effective in depressive episode and whose role in prophylactic treatment also encompasses cognitive function. While beta, left delta and theta activities are normalized with lithium, treatment response is most closely associated with basal dela activity. Lithium plasma concentration is correlated with theta activity (Silverston et al., 2005). With the addition of carbamazepine to lithium, frontal delta activity increases while theta activity is decreased predominantly in right hemisphere (Small et al., 1989).

EEG abnormality is the predictor of unresponsiveness to lithium and anticonvulsant requirement at the end of three months (Ikeda et al., 2002). On the other hand, left dominant frontal changes may predict treatment response with lithium. After 20 weeks of lithium usage relative alpha activity is decreased in right centroparietal region (Schulz et al., 2000). Among cases with non-epileptiform EEG abnormalities, cases non-responsive to valproic acid but responsive to lithium and the opposite were reported as 30–70% respectively (Reeves et al., 2003a). Unresponsiveness to lithium, carbamazepine and risperidone is associated with diffuse theta activity and high left frontotemporal amplitudes (Small et al., 1997, 1999).

Lamotrigine added in euthymia reinforces emotional stability by regulating cingulate cortex activity at the end of twelve weeks (Jogia et al., 2008). It contributes to recovery by suppressing amygdala activity in depressive episode, correlated with HAM-D (Chang et al., 2010). It ensures inhibitory control by regulating emotion and cognition in prefrontal dorsolateral cortex after four weeks of antipsychotic treatment (Pavuluri et al., 2010).

In cases with borderline and antisocial personality disorder valproate-responsive aggression rate was 36.4% whereas in non-epileptic EEG abnormality it is 25% (Reeves et al., 2003b). No consistent QEEG changes were shown in cases with subthreshold mood irregularity after 12 weeks of valproic acid treatment (Chang et al., 2010). We think that the more severely pathologic the biological projection of impairment of impulse control (here, EEG) in borderline and antisocial personality disorders, the more effective will be the specific treatment of the existing biological problem (here, aniconvulsants) on the symptom.

Psychotherapy has been shown to contribute to the normalization of IFG hipoactivity in the twelfth week (Favre et al., 2013). Awareness-based CBT has been shown to decrease right frontal beta activity (Howells et al., 2012).

In a study investigating first manic episode cases in manic episode and subsequent period of remission, right frontoparietal and left frontotemporal beta activity are found to be increased in manic episode (Güven et al., 2015). No association was shown between beta activity and YMRS scores. While right frontal alpha activity was increased in wellness period, interestingly right frontal alpha activity distinguished between psychotic and non-psychotic cases. Lower alpha activity in euthymic bipolar cases compared to healthy controls is a previously reported finding (Başar, 2012). On the other hand, increased right alpha synchronization is reported in schizophrenia-like psychoses and epileptic patients with psychotic symptoms (Canuet et al., 2011) and this could be interpreted as a parameter pertaining to postpsychotic depression.

## 2 Conclusion

Consequently QEEG harbours affective and cognitive components for the assessment of current situation in cases diagnosed with affective disorders. QEEG is more cost-effective and practical compared to other electrophysiological studies and functional imaging methods. It increases the cooperation of noncompliant patients by providing objective evidence and alleviates self stigmatization. The capability of these directly obtained data with high temporal resolution to support the diagnosis cross-sectionally is not a contribution to be underestimated. These directly obtained data with high temporal resolution contribute even more to clinical assessment in regards to treatment response monitorization. Additionaly it probably carries information about longitudinal course and residual symptoms in periods of wellness. Future studies should target depression subtypes, bipolar disorder subtypes, course features, comorbid conditions and possible differences among specific treatment algorithms.

## Declarations

### Author contribution statement

All authors listed have significantly contributed to the development and the writing of this article.

### Funding statement

This research did not receive any specific grant from funding agencies in the public, commercial, or not-for-profit sectors.

### Competing interest statement

The authors declare no conflict of interest.

### Additional information

No additional information is available for this paper.
